# News and Notes

**Published:** 1910-09

**Authors:** 


### REGULAR MEETING OF THE FULTON COUNTY MEDICAL SOCIETY

### JUNE 2d, 1910.

REPORTED BY R. R. DALY, M. D.

---

Dr. Toepel read a paper on "Sequelae of Sprains."

Dr. Lindorme said that he had treated ankle sprains by using pieces of book-board as splints. These could be easily removed while the ankle was treated with hot water spray. The recovery is quick in such cases where the ligaments are not entirely torn loose.

Dr. George N. Niles read a paper on "Dyspepsia of Old Age."

Dr. Todd said he enjoyed the paper as he does all of Dr. Niles' essays. The pendulum of change in the treatment of all diseases has swung too far; everything is changing, it is true, but it is now in direct contravention of our best methods. Osler said a true thing when he stated that professors should stop teaching at sixty. He thinks Niles did not put enough stress on the liquid diet for old people. Losing teeth in age leads us to poorly mastricated food.

Dr. R. T. Dorsey said that probably more than 66% of dyspepsia of old age is due to some organic trouble. When a person of sixty-five develops dyspepsia it is very likely due to carcinoma. Old people have reached the age of worry and they are very prone to read the mortality columns of the papers and this depressing influence possibly causes 10% of indigestion. Heart disease is a marked cause of indigestion of old age. Twenty-two people died in this town with that trouble last year. The angina pains are misinterpreted as acute indigestion, which, in itself, can not induce sudden death. In true angina cases indigestion comes first as a symptom. Dr. Dorsey's own father died of angina but was treated for stomach trouble a long time before the heart trouble was diagnosed. It is important to note the heart conditions early because life may be greatly prolonged.

Dr. Wesley Taylor said that Dr. Niles always reads an interesting paper. A prominent physician in London makes it a rule in his clinic to examine the heart when the stomach is complained of and vice versa. Old people at eighty or more have the dried-up variety of dyspepsia and the liquid foods are necessary for them. He agrees with Dr. Todd that the old should not be deprived of alcohol.

Dr. Kime said he wondered whether dyspepsia was the cause or result of trouble; a man fifty-five or so must learn that he can not keep up excessive eating. He spoke very strongly against excessive feeding and against any stimulants whatever. A doctor who advises all the food and drink a man wants when the patient has interstitial nephritis and indicanurea should have his license revoked.

Dr. LeConte said there was no special type of old age except as the ordinary types were a little changed in advancing years; often the trouble began as the patient became an old man; it grew into permanence as he passed sixty-five. Said a man or woman is very hard to manage as to diet; they "don't have to be careful." He agrees with Dr. Dorsey that death from acute indigestion does not come from indigestion alone, but that recently there has been an imprudence in eating—within twenty-four hours. A full fundus of the stomach presses against the heart which, being in fatty degeneration is thus interferred with. Catarrhal colitis often causes dyspepsia in old age together with auto-intoxication.

Dr. Niles said, in closing, if he could have heard the discussion first he could have improved his own paper and thanked the gentlemen.

### PROCEEDINGS OF THE AMERICAN PROCTOLOGICAL SOCIETY.

### *President's Address—"Undergraduate Proctology."*

BY DWIGHT H. MURRAY M. D., OF SYRACUSE, N. Y.

After thanking the society for the honor conferred upon him in making him president, he made some recommendations as to its future before taking up the formal subject of his address.

He conisdered that the American Proctologic Society stood for a high class of scientific work and the best that there is in proctology. He believed that it would be for the best interests of the society that the programs of future meetings should be made up of a symposium, or possibly two, with essays that shall treat thoroughly some selected subject or subjects, and that these papers should be written by men whose part in the symposium should be assigned to them by the executive committee. He suggested that the program should not be too crowded and that sufficient time should be given for a full discussion of every paper and subject presented.

He believed that a volume or year-book of the American Proctologic Society containing a symposium with additional papers of merit such as would be presented by experts in proctology could be made of great value to the profession and would be sought after by general practitioners. He believed that it was of the utmost importance to the society that the transaction be published yearly as it would be a decided step backward to omit the publication no matter what its cost might be.

A recommendation was also made regarding the limitations of the field of the proctologist. He believed it to be true that the ethical practice of proctology was too narrow a field in which the specialist could gain a competence. He, therefore, recommended that this society take up the question of the limit of proctology as a specialty and that it be changed to include diseases of the small intestines, in other words, that proctologists become procto-enterologists in this way every member of the speciality would be doing uniform work.

He then proceeded to take up the main subject of his address, "Undergraduate Proctolgy." He believed that the speciality was rapidly assuming the importance which is its due, in spite of the opposition it has experienced from the generai surgeons who seemed to look upon it as an unwelcome invasion of their field.

He considered that one of the most important duties of the Proctologic Society was an educational one. He hoped that with the increasing appreciation and demand for this kind of special work, that the colleges would take up the subject in a manner which its importance demands, and that if the medical colleges did not educate the profession in this branch of medicine, the members of the Proctologic Society must do it. He put forth the claim that the field of medicine and surgery is too large to admit of any man becoming an expert in all branches. This is an age of specialties and the very limitations of a specialist make an expert of him.

He believed that protologic teaching in colleges should be done by men learned in the specialty and not by general surgeons who only teach in a desultory manner, so that when the students are graduated they go forth to the practice of their profession in fully seventy-five per cent. of the cases, with little or no knowledge of this line of work.

He then proceeded to prove this point by a statistical report showing the answers to questions which he propounded in a communication to fifty of the most prominent colleges in the United States and Canada. The answers to those questions show conclusively that a very large percentage of the college faculties believe that proctology is of minor importance and that it is not necessary to give the student any special training in the subject.

In order to prove his point he found it necessary to communicate with a large number of physicians, including specialists in various branches and men who had graduated during the years from 1873 to 1905. He sent communications to these men asking them to answer certain questions which would show whether they believed they would have been better prepared for their practice and have been better able to treat their patients, if they had been given instructions in this line of work.

Ninety per cent. of the physicians answered the questions in the affirmative, which he believed told the story from the standpoint of the physician. This gave him good comparison from the standpoint of the college faculty on one hand who feel that they know the subjects in which the student should be trained at the beginning of his life work, and from the standpoint of the physician on the other hand who is in the midst of his life work. These answers show that physicians believe that colleges should devote less time to major things in specialties and surgery, and instead give their students more definite and practical instruction in proctology.

Dr. Murray then presented the questions and answers from the college faculties and physicians in tabulated form. He did not claim that the work of the eye, ear, nose and throat or any of the specialties was unimportant, but he did maintain that the time given to these specialties should be shared in a proper way with proctology, which would not detract from the importance of the older specialty but would recognize the importance of proctology. At the same time this would put the young graduate in possession of knowledge that would not only be of great value to him but of far greater value to his patients. There are certain common and important diseases in every specialty that the young physician is sure to meet and ought to be able to recognize.

He believed it to be the duty of the American Proctologic Society to foster a sentiment in the profession and among college authorities favorable to the special teaching of proctology either separately or as a branch of general surgery. He did not deem it necessary that a special chair of proctology should be created, but that a course in proctology should be provided for under the chair of general surgery.

Dr. Murray believed that it would be wis efor the American Proctologic Society to offer a prize for a substantial sum of money for the best original graduating thesis on a proctologic subject. The competition to be pen to graduting clases of any college in the United States and Canada.

In conclusion the doctor believed that the profession should offer more encouragement to specialties in all branches, especially to those who are willing to devote their time to branch which has for some reason been neglectd, as proctology has been. Thn it wuld be practically impossible for quacks and healers of various sects and isms to take advantage of our professional neglect, and use it as their opportunity to play upon the credulity and gullibility of human nature.

---

*"Review of Proctologic Literature From March,* 1909, *to March,* 1910.

BY SAMUEL T. EARLE, M. D., OF BALTIMORE, MD.

The Committee on Proctologic Literature reviewed the following papers as worthy of the attention ofthe members of the Proctologic Society.

"The Treatment of Hemorrhoids by Zinc-Ionixation," by Dr. T. J. Bokeham, which appeared in the Proceedings of the Royal Society of Medicine, May, 1909, page 135.

A paper by Dr. Herman A. Brav in the *Monthly Cyclopedia and Bulletin,* May, 1909, page 268. "The Importane of Careful Post-Operative Treatment in Rectal Operations."

A paper from the Albany Medical Annals May, 1909, Vol. XXX., by Dr. George Blumer, New Haven, Conn: "A Neglected Rectal Sign of Value in the Diagnosis and Prognosis of Obscure Malignant and Inflammatory Diseases Within the Abdomen." The sign is spoken of as the rectal shelf, which is observed on making a digital examination of the rectum on the anterior rectal wall, from two to four centimeters above the prostate gland in males. This shelf is of almost cartilaginous feel which projects into the rectal cavity. In some cases the circumference of the rectum is involved in an annular zone of infiltration, more marked anteriorly and tapering off toward the posterior wall, a signet ring stricture, as Schnitzler calls it. The summary of this paper is contained in the following:

1. In certain forms of carcinoma of the abdominal organs, notably gastric carcinoma, and in some cases of tubercular peritonitis, implantation metastases in Douglas' pouch are common.

2. These metastases impinge upon the rectum and may infiltrate its submucosa, causing a pecular shelf-like tumor on the anterior rectal wall, readily felt by the examining finger.

3. In cases of gastric carcinoma this may be an early metastasis, and occurs especially in males.

4. In such cases the primary tumor may be latent and the metastasis may be large enough to cause symptoms of obstruction. It has been mistaken at times for rectal carcinoma and has been removed as such.

5. The not infrequent occurence of this rectal shelf makes it a diagnostic and prognostic sign of a good deal of importance, and warrants the statement that in no case of obscure abdominal disease should a rectal examination be omitted.

Dr. W. I. DeC. Wheeler, in the London Lancet, March 6, 1909, gives excellent reasons for always using the abdominal route, or a combined method for excision of carcinoma of the rectum, whenever the malignant growth is three inches or moer above the sphincter.

The technic for Excision of the Rectum in Procidentia, as given by Dr. John H. Cunningham, Jr., Boston, Mass., Annals of Surgery, May, 1909, is referred to and favorably commented upon.

Dr. A. L. Wolbarst's improved rectal irrigating tube is referred to. A description of the instrument may be found in the Journal of the American Medical Association, July 31, 1909.

---

### "MALFORMATIONS OF THE ANUS AND RECTUM; REPORT OF FOUR CASES."

By Alois B. Graham, A. M., M. D., of Indianapolis, Ind.

Congenital malformations demand prompt surgical treatment. Many cases are never reported and the percentage is evidently much larger than statistics indicate. These malformations are sufficiently uncommon and interesting to warrant placing every case on record. Report of four cases.

Sase I.—White male child, born with no trace of an anus, and in whom careful dissection and exploration failed to find any trace of a rectum. Colostomy was suggested but the parents refused their consent. Child died four days later. Autopsy refused.

Case 2.—Colored male child, age five years, born with a complete obstruction of the anus by a membranous diaphragm, which was perforated by the attending physician. Examination revealed a dense stricture, almost impermeable, involving the entire anal canal. The interesting point was the presence of a hypospadias through which feces had escaped for two years. The communication between the rectum and urethra was the result of ulcerations above the stricture rather than defective embryological development. Surgical treatment was refused.

Case 3.—Colored female child, age fifty-six days, in whom examination revealed a well-formed anus and a protruding or bulging imperforate rectum. A photograph shows a pronounced distention of the abdomen the result of a fifty-six days intestinal obstruction. Posteriorly, the rectum had no attachments, and the finger could be introduced easily behind the bulging imperforate gut, through the anal canal, into a blind pouch. A fistulous opening was found in the vagina just behind the hymen. The meconium and a small quantity of feces had escaped through this opening. The protruding rectal mucosa was dissected from its attachments and excised. The rectal mucosa was then sutured to the free skin at the anal margin, except for one-eighth of an inch posteriorly. . This was used for drainage in case the blind pouch became infected.

This patient made a good recovery. At the dast examination which was thre months following operation the finger could be introduced easily into the rectum, the stools were normal, and sphincteric control was good. The fistulous opening into the vagina was closed, and the posterior rectal mucosa was firmly united to the skin at the anal margin. With the exception of an abdomen, which seemed to be a trifle prominent for one of its age, the child appeared normal.

Case 4.—White child, one of twins, age forty-two hours, in whom examination reveaeld an imperforate urethra and no trace of a anus. Penis and scrotum were well developed, but neither testicle could be palpated. Careful dissection and exploration failed to find any trace of a rectum. A two inch incision was made in the median line just above the pubis, but no bladder could be found. Decided to perform a colostomy or sigmoidostomy. A portion of what was supposed to be the sigmoid was opened and a large quantity of meconium escaped. Exploration revealed a pouch which appeared of much larger dimensions than a normal colon or sigmoid should be. Operation was completed, and yet our inability to find the bladder made the case a hopeless one. Child died twenty-four hours later. At autopsy no bladder was found. The entire large intestine was removed. This case is of interest from the point of view of defective development. The pouch-like termination of the intestine might well be termed a monstrosity. The writer is inclined to believe that it is one of those rare cases in which the colon or sigmoid opens into the uterus. While the local examination revealed a male child, with the exception of being able to palpate the testicles, the examination of the speciment removed at autopsy reveals marked evidence of the female generative organs. This child was a transverse hermaphrodite—namely, one in whom the external genitals seem to be of one sex and the internal of the other. Report of examination of specimen states that the pouch-like termination of the intestine is formed of three organs; namely, the bladder, uterus and rectum. (Specimen shown.)

---

### "THE USE OF QUININE AND UREA HYDROCHLORIDE AS A LOCAL ANESTHETIC IN ANO-RECTAL SURGERY."

By Louis J. Hirschman, M. D., of Detroit, Mich.

Dr. Hirschman presented to the Society, a report of his work with quinine and urea hydrochloride as a local anesthetic in ano-rectal surgery. The cases operated upon were as follows:

Acute Thrombotic Hemorrhoids 10; Internal Hemorrhoids 22; Interno-External Hemorrhoids 7; External Hemorrhoids 10; Fistula-in-ano 14; Abcess peri-anal 7; Fissura-in-ano 7; Excision of Scar Tissue 3; Ball's Operation (Pruritus ani) 2; Hypertrophied Papillac 16; Inflamed Morganian Crypts 4; Total 102.

He reported perfect results as far as operative anesthesia was concerned in every case, and in but seven cases was there any post-operative pain. He usese the one per cent. solution of quinine and urea hydrochloride in all of his cases of ano-rectal surgery, where suturing of the skin is not required.

The technic of adminstration as employed by Hirschman is the same as that used with weak solutions of cocain and eucain. He describes this technic in detail. He believes that the substitution of quinine and urea hydrochloride for any of the other anesthetic salts hitherto employed will be found eminently satisfactory in all cases of ano-rectal surgery, where suturing of the integument is not required. He sums up its advantages over the other anesthetic drugs as follows:—

First: It is soluble in water.
Second: It can be sterilized.
Third: It is equal to cocain in anesthetic power.
Fourth: It is absolutely non-toxic.
Fifth: It has a pronounced hemostatic action.
Sixth: Post-operative anesthesia lasts from four hours to several days. d
Seventh: It is inexpensive and most always available.

(TO BE CONTINUED.)

